# The Medical College Craze

**Published:** 1897-08

**Authors:** 


### The Medical College Craze.—A Circular Letter.

*To the Medical Profession of Texas:*

Gentlemen:—No doubt many of you have seen from the daily press that attempts are being made to organize a medical school at Dallas. A respectable portion of the physicians here, who hold no higher motive than honor to our noble profession, whose mission has ever been to unfold hidden truths and to bless mankind, have already entered their solemn protest against the above named organization, and we think it wise and judicious to give you our reasons and ask your co-operation. No sickness no doctor; do doctor no medical school. This sentence tersely and succinctly sets forth the right and true relation between the people, the doctor and the school. The only foundation at all for the existence of the medical profession as a separate body is the fact of human suffering, and the only foundation for the existence of the medical school is the necessity to fit men to prevent, alleviate and cure human ills. Upon the right conception of the relationship thus set forth between the people, the doctor and the school, depends all that there is of honor, dignity and true service in our noble profession, and constitutes the foundation stone upon which our high calling is built, and ought forever to separate us from the spirit of commercialism, which, in these latter days, holds nothing sacred and attempts to put its trade mark upon all things human and even divine. "Quae omnibus prosunt," not "cui bono," should be inscribed upon our banner. A sincere and honest desire to serve our fellow-men being the governing motive of the true physician, should not the same noble ambition to serve their day and generation animate and control men engaged in the high vocation of teaching? When the question of establishing a school of medicine is discussed, the real honest question is not, will it pay; will it be good for the teachers, will it bring money to their purses, or will it advertise the professors, but is it best for the community and the profession; will it add usefulness or dignity to the already over-crowded profession? We have already 140,900 to 150,000 doctors in the field, and the diploma mills now grinding day and night with an annual output of 15,000 to 20,000 more. Isn't it time to pause and reflect; can there be any earthly excuse for even one more college? Would it not be infinitely better, if we could do so, to shut up and obliterate and abolish from the face of the earth 75 per cent of those colleges already in existence? Is it not a fact, that by the multiplicity of medical schools the doors have been thrown wide open to uneducated and unfit men, men unprepared to acquire or successfully pursue a learned profession, taking them from the shop, the field and the ranch, where they might have been producers of something valuable to themselves and their kind? Who amongst us does not have personal knowledge of those unseemly scrambles for students? The bars must be lowered for their matriculation and the bars must be lowered for their graduation. So we are forced to the conclusion that the multiplication of medical schools is the largest factor in the deterioration of our profession, in the loss of the dignity, morale and high tone that once characterized it.

But there are other objections of minor, but still important bearings. In addition to the unseemly scramble for pupils, which affects the country at large, here comes this objectionable feature, especially to the local profession, viz: the keen, still hunt for clinical material. And the preserves of the honest, hard-working non-professor are unscrupulously poached upon in order that *the professor* may swell and strut and look wise things if he doesn't say them. Indeed, to such an extent do the private practitioners in some of our larger cities suffer that they are forming protective leagues among themselves to prevent these encroachments. Horrible to relate, many of these alma maters live and thrive and have their being from the blood of their offspring. They covenant and sell and deliver the diplomas, and then straightway, if the new-fledged doctors dare settle under the shadow of their walls, begin to filch their patients. Oh, sweet alma mater! We are glad to say, however, that here and there in our broad land, there are noble institutions, amply endowed, surrounded and crowded with clinical material, with high requirements for matriculation and graduation, where the earnest student can get every advantage, and if he but comply with the requirements, can leave their halls with the assurance to himself and the promise to the people of at least a useful if not brilliant career. It is simply impossible to establish and maintain a school of this grade at Dallas. In the first place, the necessity for the school does not exist. We are in less than a day's ride of fifty schools—in less than forty-eight hours of 200 more—of all classes and grades—from Cheap Johns up to the highest and noblest institutions that grace and bless any land. Then, again, it is simply idle to claim for Dallas the required clinical material. In the history of all professions there comes a time when the good and true men are expected to stand firm. That time is upon us now. Gentlemen, we are in the midst of battle. We heartily implore you to join us by withholding your countenance and support from the establishment of any new colleges. We would welcome a kind word of approval from medical associations and from private practitioners.

S. Eagon,

W. R. Wilson,

S. G. Thruston,

V. P. Armstrong,

B. F. Church,

A. C. Graham,

O. L. Williams,

J. C. McMahon,

Samuel W. McJunkin,

Willis R. Smith,

R. W. Allen,

Wm. Morrow,

